# The Transition of Children Living With Congenital Heart Disease to Adult Care

**DOI:** 10.7759/cureus.50179

**Published:** 2023-12-08

**Authors:** Ashu Tyagi, Tushar Sontakke

**Affiliations:** 1 Department of Medicine, Jawaharlal Nehru Medical College, Datta Meghe Institute of Higher Education and Research, Wardha, IND

**Keywords:** follow-up, self-care, multidisciplinary care, transition, adult congenital heart disease, congenital heart disease

## Abstract

The article explores the significance of the timely transition of a child living with congenital heart disease (CHD) to adult care and the role played by multidisciplinary care. Due to recent healthcare advances, more children with CHD survive to adulthood without surgical intervention. This survival is mainly due to the lesion being compatible with life and its management being done medically. However, further management requires meeting the child's needs and helping him transition to become a healthy, independent adult with almost equal life expectancy as his counterparts. The article reviews the comprehensive framework of transition through multidisciplinary care. Highlighting the necessity of training physicians to acquire expertise in the management of CHD is a foundational aspect of this review article. Introduction to transition requires assessment of the child's needs through all phases of life and informative counseling of both parents and child. It highlights the approach to educating patients and families with the knowledge to safeguard compliance. Multidisciplinary collaboration from various fields such as cardiology, pediatric physiatrist, nursing, and psychology has been stressed. Patients also need to cultivate skills in self-management and independence and be educated to comprehend their condition, including the potential health issues. This collaborative and multidisciplinary process necessitates the cooperation of patients, families, and the adult congenital heart disease (ACHD) team. Emphasis has been given to individualized counseling for girls to address their sexual health. The article also highlights the possible obstacles and how to tackle them to improve healthcare adherence. Timely transition and follow-up can be measured using various tools or through indices measuring the quality of life and average life expectancy. The global patterns of transition to ACHD care have also been emphasized, as well as the need for research studies to develop reliable indicators for assessing transition success.

## Introduction and background

The advancement in healthcare has led to the successful management of children with congenital heart disease (CHD), leading to an increased number of adults presenting with adult congenital heart disease (ACHD). CHD is one of the most common congenital abnormalities seen in children [1]. Among this population, a large group of adults never underwent cardiac surgery because of the benign asymptomatic nature of the defect, the lesions remained undetected, medical services were not accessible, or the defect was inoperable. The lesions most commonly compatible with survival to adult life without surgical intervention are ventricular septal defects, atrial septal defects, mild pulmonary stenosis, and mild aortic anomalies [2].

The upward trend in presenting ACHD patients to healthcare poses a unique challenge. As these individuals age, their medical needs evolve, necessitating the development of specialized transition programs. The term "transition" refers to the shift from child-centered medical care to care that meets the child's distinct medical and emotional needs as they enter adulthood. Therefore, transitioning from pediatric to adult-centered care is essential [3]. The ACHD patients demand specialized care, requiring healthcare providers to possess expertise and training to deliver appropriate treatment. Transitioning care involves a systematic and ongoing progression from family-centered pediatric care to patient-centered adult care. To facilitate this transition effectively, patients must develop self-management and self-advocacy skills to assume greater independence and medical responsibility to comprehensively understand their condition, including the potential coexisting health issues. This multifaceted process necessitates collaboration between patients, families, and the ACHD team [4]. In clinical practice, various models can be applied to enhance the transition process for youth with CHD based on their individual needs. Overcoming the barriers to transition is also an essential part of the process. These barriers must be identified, and appropriate ways to tackle them must be considered while evaluating transition programs; short - and long-term impacts present challenges, such as different goals and definitions of success that coexist for each individual [5].

## Review

Methodology

**Figure 1:**
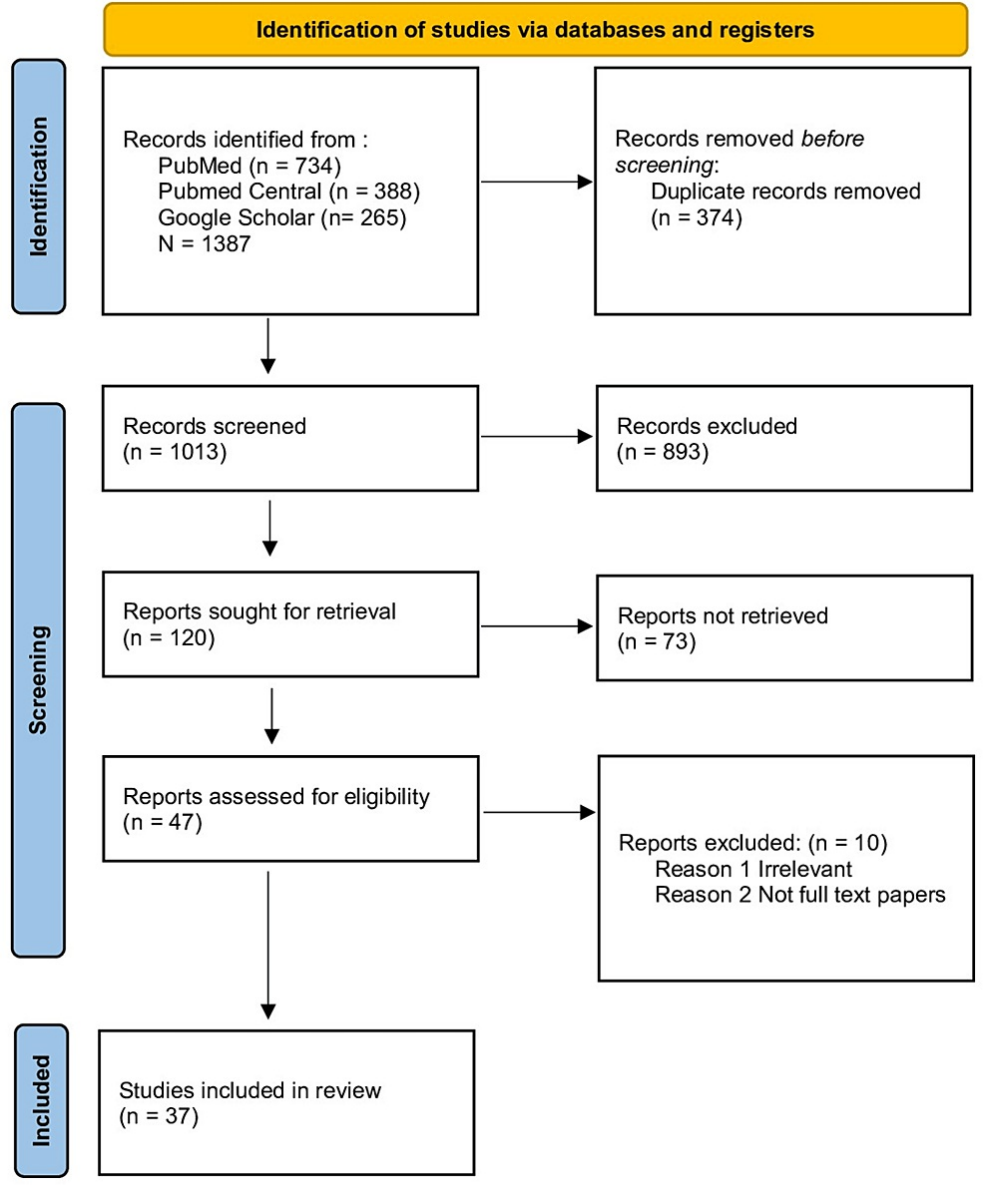
PRISMA 2020 flow diagram for the systematic review PRISMA: Preferred Reporting Items for Systematic Reviews and Meta-Analyses; PMC: PubMed Central

The transition of a child with CHD to adult care was methodically reviewed using a literature search. The combinations and keywords used were self-care, multidisciplinary care, transition, adult congenital heart disease, follow-up, and congenital heart disease. Using databases such as PubMed, Medline, and Google Scholar, an extensive literature search was done and cross-referenced to identify studies relevant for inclusion in the review. Articles from the last 18 years were used in the search. Reference lists of relevant publications and review papers were reviewed in addition to electronic database searches to find more studies. The selection process for the research that satisfied the inclusion criteria included experimental studies, observational studies, meta-analyses, and systematic reviews that looked at the transition to adult care of a child with CHD. The inclusion of only peer-reviewed, published articles was taken into consideration. Titles, abstracts, and full-text publications were evaluated independently, and any inconsistencies were settled by discussion and agreement. The extensive literature search thoroughly examined the transition to adult care of a child with CHD, which aimed to ensure the inclusion of pertinent research. The method used to choose our study's papers is shown in Figure 1.

Approach for transition

The approach to transitional care needs to be optimally started in early adolescence and extended into emerging adulthood. Based on the child's age group, understanding, and participation regarding their health, the transition program can be divided into three phases: pre-transition, transition, and post-transition [5]. The pre-transition phase is the most significant and should commence during early adolescence with specific interventions in mind that need to be implemented at certain ages to achieve the pivotal milestones. However, since the need for personalized care and flexibility in the timing of milestone achievement is different for different individuals, the steps in transition may vary according to them. Considering the child's needs, the interventions should be organized systematically to form a well-structured transition program [6].

Pre-transition Introduction

The transition process should be introduced to the child and his family around 12 years by integrating it into a scheduled outpatient visit or through an introductory letter sent to the parents [6].

Assessment of Needs and Progress

A comprehensive assessment tailored for the adolescents should be conducted through an interview called Home, Education, Activities, Diet, Drugs, Depression, Sex, and Safety (HEADS) around 14 years of age [7]. It offers insights into the adolescent's lifestyle and living circumstances, shedding light on strengths and areas needing attention. While not exhaustive, these questions guide the interview process. Monitoring the progress of needs and capacities, often referred to as transition readiness, is critical during the transition phase [8].

Education and Counseling

Based on the interview, data collected should be employed via communication styles suitable for adolescents. They should be actively engaged in education and counseling sessions. These sessions should cover critical subjects, including CHD management, the necessity for lifelong medical follow-up, reproductive considerations, physical activity, endocarditis prevention and prophylaxis, and healthy lifestyle choices. Patients and families should also be sensitized about sexuality, contraceptives, and religious perspectives. Strategies aiming at goal-setting and empowering patients should be formalized [8].

Developing and Collaborating on a Plan

The assessment outcomes and the results of counseling sessions should be documented within a comprehensive transition plan. This plan is an evolving resource that gets progressively completed throughout the transition process. It should encompass critical components, including patient and caregiver information, the requirement for surgical support and care, a concise medical status report, support needs, recommendations concerning physical exercise, medication, endocarditis prevention, follow-up, reporting from the HEADS assessment, transition goals, available assets, and identified needs as communicated by the patient [8].

Facilitating Contact With Peers

These children with chronic conditions often need opportunities to connect with others facing similar situations. Whenever feasible, facilitating such peer interactions is vital. Centers should collaborate with youth ambassadors and local associations to encourage peer support and organize annual events dedicated to adolescents. In cases where societal stigma hampers in-person gatherings, fostering peer connections through social media platforms can be encouraged to ensure meaningful support networks [8].

Introducing the ACHD Team

A pivotal step in the transition process involves acquainting patients and their families to the healthcare team, the outpatient clinic settings, and the procedures for each visit. This initial encounter significantly influences the success of the transfer. This should occur through a visit to the specialty clinics, interactions with the ACHD team, distribution of brochures or flyers, or even virtual presentations using slides or video [6].

Transfer to Adult Healthcare

In regions where these facilities are accessible, referral to the ACHD team is recommended rather than just informing them about the closest center. A letter for transfer with a medical summary written thoroughly needs to be provided to facilitate this transfer. Advocates have proposed that every patient visit a specialized ACHD center at least once. In geographical areas without such specialized centers, these individuals must be directed to clinicians with some ACHD care training [8].

Monitoring Continuity of Care

To ensure consistent follow-up, the pediatric team should establish a scheduled time for the next appointment with the ACHD team and send an invitation to the family. In cases where they fail to attend the initial transfer appointment, the team must implement a system for reminders, as future compliance is based on this first appointment. Methods like short text message reminders have improved healthcare appointment attendance. In missed appointments, dedicated administrative personnel should remain attentive to ensure they receive another invitation for rescheduled appointments [8].

Guidance for Parents

**Figure 2:**
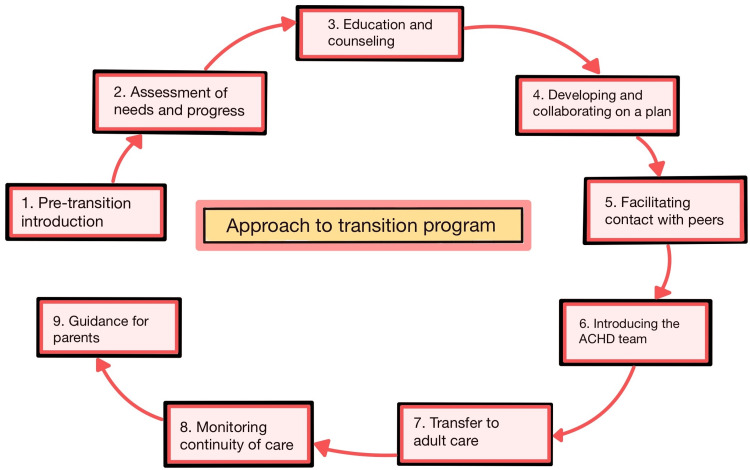
Summary of approach to the transition program Self-created ACHD: Adult congenital heart disease

The transition process often poses more significant challenges for parents than adolescents. Parents need to adapt to evolving their roles. Offering specific support to parents during the transition phase helps mitigate parental stress and anxiety and improves transition outcomes. Parents should be informed in accessible formats and languages suitable for them, considering their cultural diversity and health literacy levels [9]. Figure 2 summarizes the approach to the transition program.

Transition time

The ideal time for transition may vary according to the needs of the individual. However, the proposed time for the initial shift is considered between the ages of 12 and 14. During this age, the child is expected to manage a doctor's visit autonomously for a portion of the appointment, formulate one question for each medical session, become familiar with reaching out to and connecting with healthcare experts, and acquire knowledge about their cardiac condition's name as well as the names of their prescribed medications [10]. In the intermediate phase, from ages 15 to 17, the individual should take on the role of primary communicator with the physician during appointments and independently schedule their medical visits. They should also grasp the process of filling prescriptions, offer a concise three-sentence overview of their cardiac background, manage their medications without assistance, and comprehend medical urgencies and emergencies [11].

In the final transition phase between 18 and 21, the individual is expected to manage doctor visits and appointment scheduling autonomously. They should also become acquainted with an ACHD healthcare specialist or acquire the skill to locate one and a corresponding facility. Additionally, they should recognize the need for ongoing care and achieve a health-conscious lifestyle [11]. Irrespective of the transfer age, this process must not culminate solely after the shift to adult healthcare. The ACHD team must have specialized knowledge in transitional care, aiming to facilitate a smooth transformation and assist patients in assimilating into adult healthcare and life [11].

Role of multidisciplinary care

Children with CHD have special needs, which, if not fulfilled, may lead to certain handicaps. These can be prevented through proper intervention by healthcare providers in multiple disciplines in the transition program [5]. To begin with, pediatric physiatrists specializing in rehabilitation care aim to facilitate functional recovery, attain age-appropriate self-sufficiency, and provide optimal developmental outcomes that enhance the overall quality of life. They do so by devising treatment strategies and offering suitable medical interventions. Additionally, they provide educational support, oversee care coordination, and prescribe rehabilitation equipment for the child's benefit [12].

Cardiologists and cardiovascular surgeons are responsible for assessing cardiac health and granting approval for exercise-based rehabilitation. They evaluate exercise capacity, design tailored exercise training programs, and provide directives concerning specific preventive measures [13]. The nursing department plays a pivotal role in delivering skilled nursing care. They conduct cardiac evaluations, gather essential data, provide comprehensive education on symptom identification, and aid in coordinating the efforts of the multidisciplinary teams. They ensure the patient and their family comprehend and adhere to all aspects of care. Exercise physiologists oversee and guide exercise programs involving responsibilities like monitoring heart activity. They collaborate within the team to ascertain the most fitting fitness regimen for every patient. Speech-language pathologists specialize in evaluating and treating speech, language, feeding, and swallowing disorders, and they contribute to the recovery of age-appropriate cognitive functions, speech abilities, language skills, social communication skills, reading proficiency, and feeding and swallowing abilities. They actively facilitate effective communication between patients, caregivers, and medical providers [13].

Physical therapists are crucial in maintaining and enhancing the best possible physical performance for healthy well-being and overall quality of life. They also aid in preventing and advancing deficits resulting from heart-related concerns. Occupational therapists focus on reinstating functional skills and daily living activities, empowering children to attain independence in home, school, and community environments. Through training and guidance, they educate children and families about managing and enhancing cardiac health as part of their daily routines [14]. Psychologists and neuropsychologists specialize in evaluating and treating a broad spectrum of neurodevelopmental, neurocognitive, and psychiatric conditions. They conduct assessments to guide treatment planning and provide support within educational settings. They offer psychoeducation and psychotherapy for children and families navigating challenges related to medical conditions. Moreover, they provide cognitive rehabilitation and academic support where necessary [3].

Tackling the obstacles to transition and follow-up

The loss of follow-up of the child during adolescence is the primary risk factor leading to the failure of the transition process. The discontinuation usually begins at an early age and includes all untraceable patients. These CHD patients may present to the Emergency Department with severe worsening of their clinical state, which may even progress to irreversible health outcomes or mortality [15]. The health determinants affecting the social ones are low socioeconomic status, no health insurance, less educated parents, housing difficulties, ethnic or transport, connected missed examinations, and loss of follow-up. Thus, evaluating these social determinants through appropriate assessment, referral, and social services can reduce the discontinuity. Transferring patients to the cardiology ward when they are too young may lead to overbooking cases [16]. It may lead to a lack of time to address multiple instances to maintain continuity of care. The problem occurs because of the number of practicing healthcare professionals, which needs to be increased. It is necessary to set up more ACHD programs to train cardiologists and other healthcare professionals required for a multidisciplinary approach, including the nursing staff [17]. Administrative staff for managing databases and records and performing follow-up calls and procedures are also necessary. Proper handing off of the patient from pediatric cardiologists to ACHD specialists should reduce the risk and be conducted at least for patients registered in tertiary care centers [18].

Another factor highlighted was that some CHD patients were told that further evaluation was not needed. Lack of communication leads to issues with drug orders, delays in discharge, and no clarifications regarding following follow-up dates, leading to frustration and contributing to the negative image of the healthcare system. Insufficient knowledge of their condition and associated needs also leads to a loss of follow-up [19]. The lack of knowledge may be regarding the medical problem, lifestyle, monitoring their care, and taking self-care in patients and parents. CHD patients represent significant stress-triggering conditions due to prolonged hospital stays or economic factors. These can be exacerbated during the transition process and thus need to be addressed through counseling by psychologists of both parents and children. A way to retrieve ACHD patients can be through developing a digital health record database to maintain their documentation and follow-up dates and a possible scoring system to assess and group them. All this necessitates a transition program that ensures planned and coordinated transfer of care [20]. 

Transitioning ACHD patients must occur even in regions where these facilities are unavailable to maintain ongoing care and support, as these patients require lifelong follow-up facilities to cater to their needs. This can be achieved through telehealth services, allowing them to consult with expert clinicians virtually. This can help them to have routine checks regularly and get consultations. Primary healthcare physicians and cardiologists can also play a role by working with ACHD specialists to guide them [21]. Patients should be encouraged to undergo regular monitoring through echocardiograms, blood tests, and other essential tests to detect issues early. Healthcare providers must be trained to handle possible cardiac emergencies by developing emergency action plans for the admission of patients to the hospital. The ACHD patients should be encouraged to join support groups that can help impart knowledge to them. Parents must work with the local hospitals and organizations to advocate for improved ACHD services. They should be encouraged to seek ACHD care even if it takes long travel so that they can consult the specialists occasionally and stay updated on their condition. Thus, even if ideal conditions for transition and continuity of care are unavailable in the region, the child and parent must be encouraged to receive a comprehensive group through telehealth, collaboration with local healthcare physicians, support networks, regular monitoring, advocacy, and self-management [22].

Individualized sexual health and contraceptive counseling for girls

Counseling of girls attaining puberty must be started early as the average age of menarche in girls is around 12 years of age. The ACHD clinician should be knowledgeable enough to advise both the girl and her caretakers about the physiological phenomena of menstruation and what to expect from her first menstrual cycle and regarding the standard patterns, average flow, regularity, healthy cleaning habits and role of diet and physical exercise, in maintaining hormonal balance. The clinicians should also make the girl understand the potential health issues and encourage them to have regular follow-ups for proper clinical evaluation. They should also be advised to take care of their mental health as these factors also lead to hormonal and menstrual irregularities [23]. 

Healthcare professionals should emphasize discussion of sexual health openly and regularly with adolescent girls. They should be introduced to a healthy sexual lifestyle with guidance regarding the available birth control measures that can guarantee their protection from sexually transmitted diseases and unwanted childbirths. There should be individual counseling sessions focusing on the different needs of each girl with CHD. While advising them on the contraceptive of choice, two factors must be kept in mind regarding the efficacy and safety of the heart. The options for contraception are discussed. Condoms and diaphragms are barrier methods that are safe for all but have a setback in effectiveness due to user-dependent factors. Estrogen-containing pills are also a considerable choice with few side effects but have an increased risk of blood clots, making them unsuitable for women with certain CHD conditions like pulmonary hypertension, artificial valves, and rhythm problems. Progestin-only methods are safe for all heart conditions but may lead to irregular bleeding, which can be a drawback. Injectable and implantable forms are highly effective and safe for all women with ACHD, and they can be discontinued when a patient wishes to become pregnant with minimal side effects. With proper planning, the support of a partner, and guidance from ACHD specialists, women with ACHD have access to birth control methods that can be safely employed to meet their unique needs [24].

Self-role of the patient

To ensure compliance, the shift from pediatric to adult healthcare should involve a preparatory transition phase during adolescence. Within this phase, young individuals should be knowledgeable about their health and cardiac conditions to take on the responsibility for their healthcare gradually. Embracing healthy behaviors can contribute to reducing the likelihood of encountering late complications. The transition process forms part of a broader developmental phase encompassing puberty, which brings about physical, psychological, and emotional changes. It is a time when these individuals are exploring their identities and beginning to make decisions regarding education and career paths [25]. The adolescents may often feel excluded and uninvolved in the preparation process before the transfer. This sense of exclusion could elevate the risk of losing follow-up care after transitioning to adult healthcare. Therefore, fostering active inclusion and engagement during preparation is crucial for maintaining a continuum of care. The routines surrounding the transition process vary across hospitals. In specific CHD centers, these individuals are acquainted with the adult care environment and caregivers before their transfer to the grown-up CHD clinic [26]. The age of the adolescents played a role in shaping discussions. Older adolescents exhibited more interest in transition planning, information acquisition, and the transfer itself. Conversely, younger participants frequently expressed frustrations concerning communication and managing their condition [27].

Adolescents should receive information about their diagnoses and medications, which should be continually reinforced to prevent misconceptions. Explicit information concerning factors that could impact health, such as alcohol, smoking, physical activities, and limitations, should be mentioned during counseling. This type of information is often complex and contradictory, requiring further understanding [28]. Encouraging adolescents to participate in their care involves teaching them how to administer their medications and familiarize themselves with the complex medication names. Adolescents should be encouraged to ask physicians to address questions directly instead of their parents. This approach can help build a sense of ownership and promote reflection about their health and self-management [29]. Transforming the physician-patient interaction into a dialogue rather than a one-sided conversation with questions directed only at the parents is essential. Adolescents should comprehend the importance of ongoing medical follow-up as they transition into adulthood [30]. Throughout this process, parental support remains crucial. Learning to communicate about their condition with others may pose challenges for some individuals. Thus, participating in group interactions can facilitate the sharing of opinions and experiences. Engaging with peers in these settings can make discussing the transition process more comfortable and empower adolescents to play an active role in their healthcare journey [31].

Indicators of successful transition

The primary goal of the transition is to improve the QOL of patients. This can be measured in terms of reduced hospitalization and general physical and mental well-being, which can help the patient maintain social and peer relationships. They show increased adherence to healthcare, including medications, follow-up, and lifestyle modifications. Regular attendance at the ACHD clinic is also an indicator of successful transition. The health outcomes can be measured by assessing the cardiac function, disease progression, and absence of any complications. The transition process must help the individual develop skills in self-management to improve autonomy in taking medications, asking questions, and making appointments. This can help them build a positive attitude and confident self-image that can also help them build better social relationships and independence. The improvement in psychosocial QOL enhances the individual's knowledge to adopt healthier lifestyle practices related to diet, exercise, birth control measures, and pregnancy safety. Transition readiness among adolescents can also be measured using various tools, such as the Transition Readiness Assessment Questionnaire (TRAQ), which measures self-management, self-advocacy skills, and responsiveness to interventions. The development of these measures allows structural improvements in the transition programs and compares efficacy among different ACHD centers [6].

Limited research has been conducted concerning the anticipated life expectancy of individuals with ACHD. Despite the increased mortality rates observed in those with moderate to severe CHD with a life expectancy of up to 50-60 years, most of them perceive their life expectancy to be within the normal range [32]. Individuals with CHD may need to understand their potential long-term outcomes comprehensively. Cardiologists could potentially exhibit a more pessimistic viewpoint, resulting in a more negative estimation of life expectancy [33]. Understanding life expectancy is significant for various aspects of life, such as future planning, adherence to therapeutic measures, and timely discussions about end-of-life (EOL) matters and advance care planning [34]. Notably, individuals with very mild CHD often possess life expectancies similar to those without the condition. Those who believe their life expectancy is diminished are more open to cardiologists addressing EOL topics, improving the long-term complications [35].

Global patterns in ACHD care transition

Assessment of patterns of patient-reported outcomes in adults with congenital heart disease-International Study (APPROACH-IS) is a study spanning 15 countries that evaluated health outcomes and QOL in adults with congenital heart disease. Using the Linear Analog Scale (LAS) and Satisfaction With Life Scale (SWLS) as tools for measurement, taking the median age of the 32-year-old study group revealed generally positive QOL but with significant variation. The median LAS score stood at 80, with 10% reporting scores below 50, signifying poorer QOL. Factors like older age, unemployment, unmarried status, and higher New York Heart Association (NYHA) functional class correlated with poorer QOL. Australian patients reported the highest QOL, surpassing those in Japan, the country with the lowest reported QOL, by ten points. This variance among countries requires further investigation to understand the disparities [36]. Interestingly, previous studies comparing ACHD to healthy controls yielded surprising results: ACHD displayed better QOL due to their positive outlook, healthier lifestyle, and adeptness in adapting to their condition. This perspective suggests that investing in resources for this population is worthwhile [36].

Various tools, like TRAQ V4.0, assess adolescents' readiness for transition. A European study across 96 congenital heart disease centers found that nearly 90% offered a structured transfer, and only 41% provided transition programming. However, a recent meta-analysis highlighted that transition programs significantly decreased care gaps compared to standard care (12.7% versus 36.2%). Implementing assessment measures improves organizational functioning, evaluates program effectiveness, and establishes benchmarks for comparison [6].

The effectiveness of transition programs extends beyond transfer rates, as shown in a study in England. It revealed high transfer rates to specialist ACHD services for severe and moderate lesion patients by age 22, with minimal loss to follow-up (1.3% for severe, 6.0% for moderate). England had lower loss-to-follow-up rates than Canada and the USA, indicating potential gaps in care that could impact long-term outcomes. Patients who didn't transfer received fewer necessary procedures missing routine interventions offered in active follow-up [29]. Examining two care models-vertical (within one institution) and horizontal (split between pediatric and adult institutions)-the study found that patients from the horizontal model were less likely to transfer by age 22, irrespective of complexity. Among patients aged 16 to 22, only 1.6% died, with 42 passing away without transfer to ACHD. Although CHD patients now have life expectancies closer to those of the general population, timely transfer to adult services is crucial for adolescents to build relationships with their future care team [29].

Ongoing research aims to create dependable indicators for assessing the successful transition of CHD patients to adult care. A recent pioneering European study developed 12 evidence-based Quality Indicators (QI) tailored for patients, with eight aligning with transitional care components for better evaluation. The remaining four emphasize the need for a structured transition process via written policies. Collaboration among healthcare experts and patient representatives ensured consensus on their relevance. These QI guide centers in planning transitional care services and serve as a basis for discussions with policymakers. Integrating these QIs into transition programs has the potential to ensure adolescents receive specialized, high-quality care [37]. Table 1 summarizes all the studies involved in the review article.

**Table 1:** Summary of all the studies involved in the study Self-created

Authors	Year	Findings
Gurvitz et al. [1]	2016	The article discusses congenital heart disease as a common birth defect and the role of recent advances in the management of children to adulthood. However, the study needs more data regarding their survival, epidemiology, and long-term outcomes. It summarizes the areas of congenital heart disease-related complications, and high-priority subtopics regarding the disease have been identified.
MacGillivray et al. [2]	2019	The article summarizes that the number of adults with congenital heart disease is growing rapidly due to advancements in medical care. However, these individuals face unforeseen long-term complications despite childhood treatments, which have been discussed as such. Leading experts have provided insights into the new diagnostic modalities, surgical techniques, and their management strategies for evolving care.
Burström et al. [3]	2017	The article highlights the significance of preparation of a child with a congenital heart for transfer to adult care in terms of acquiring sufficient knowledge, becoming a participant in their care, the role of parents, and peer communication.
Stiller et al. [4]	2023	The article discusses congenital heart disease epidemiology. It highlights the management of survival of adults with this condition, shedding light upon the long-term complications, prophylaxis for endocarditis, counseling in pregnancy, and the role of the multidisciplinary model of care.
Seidel et al. [5]	2020	The article highlights the role of primary care physicians in adults with congenital heart disease. Adequate long-life specialized care is an essential determinant of their long-term survival. It examined the healthcare facilities in Germany where specialized care is available, and cardiologists are general in individual practices and centers. The results show that there are still many deficits in care due to insufficient healthcare facilities, loss of follow-up, and less awareness.
John et al. [6]	2022	The article focuses on socioeconomic factors to be considered during the transition to adult care of a child suffering from a congenital disease, designing a structured program, the role of multi-disciplinary care, causes of loss of follow-up, and the development of self-management skills in patients.
Katzenellenbogen [7]	2005	The article discusses the Home, Education, Activities, Drugs, Suicidality, and Sex interview that covers all the aspects of the development of adolescents to focus on holistic development. It has been expanded to include eating and safety.
Udholm et al. [8]	2023	The study evaluated the risk of infertility in men and women with no congenital heart defects compared to those with congenital heart defects. It summarized that the risk of infertility in adults with simple or moderate congenital heart disease was not increased.
Moons et al. [9]	2021	The article highlights the need for transition of a child with congenital heart disease while stressing the different transition program models that can be employed to meet the needs of the child. It also discusses the composition of the adult healthcare team and empowering the patient and their family to ensure improved compliance with the program.
Burström et al. [10]	2019	The article uses a questionnaire to evaluate the transition readiness of adolescents with lifelong conditions to adult healthcare. It describes transition readiness in adolescents and their parents and correlates the results. Higher scores were associated with a better level of preparedness.
Helm et al. [11]	2017	The study evaluated the transfer of children suffering from congenital diseases to adult care in Germany. The results indicated that most patients were treated by medical specialists and at adult congenital heart disease centers, with only a tiny population lacking this care, which needs further investigation.
Hsu et al. [12]	2021	The study conducted in New York provides data on the healthcare usage trends and related expenses in transitioning to adult care of children suffering from congenital diseases of the heart. It highlights the importance of a well-framed program to improve cardiac care with cost-effectiveness.
Mutluer et al. [13]	2018	The article highlights the multifaceted nature of adult congenital disease treatment, summarizing the vital points in managing disease with probable complications and long-term outcomes.
Tikkanen et al. [14]	2023	The article discusses the role of multidisciplinary teams with different expertise in nursing, cardiology, neuropsychology, feeding and occupation, and exercise training in hospital settings.
Moore et al. [15]	2022	The study evaluates the obstacles responsible for loss of follow-up, identifies the missing congenital heart disease patients, and assesses the success of interventions while transitioning children with congenital heart disease to adult care.
Hasan et al. [16]	2023	The article introduces a public health framework initiated by the Global Initiative for Children’s Surgery Cardiac Surgery group, which aims to establish services in developing countries that address the substantial global burden of these children by suggesting a framework that integrates health systems and ensures that each level adheres to good quality of care.
Jackson et al. [17]	2015	The study examines the risk of developing life-threatening complications in children with congenital heart disease while transitioning to adulthood by assessing awareness of these children about future health risk and their health behaviors.
Gaydos et al. [18]	2020	The study, through a survey in Georgia and New York, evaluates the perspective of parents of children with congenital heart disease regarding the transition process. It also introduces their common problems regarding health insurance coverage and their relationship with medical care professionals. Top of Form
Mackie et al. [19]	2019	The study discusses the transition of a child suffering from a congenital heart to adult care in Canada, explaining the collaboration between different medical disciplines, the role of ideal time in transition, preparing adolescents for self-management skills, and the role of parents in a successful transition program.
Lee et al. [20]	2017	The article signifies the function of nurses in a multidisciplinary model of transitioning children with congenital heart disease to adult healthcare. The effectiveness of the transition program is assessed through randomized trials to improve survival outcomes.
Borrelli et al. [21]	2023	The article highlights the role of telemedicine in treating children living with congenital heart diseases. It highlights the need for telemedicine in their care and recognition as a special-needs group.
Bassareo et al. [22]	2023	The article discusses the need for regular monitoring of individuals living with congenital heart disease throughout their lives. It reviews the possible causes of loss of follow-up, especially during the transition to adult care, and strategies to prevent these.
American College of Obstetricians and Gynecologists’ Committee on Adolescent Health Care [23]	2020	The article highlights the importance of a healthy reproductive lifestyle in females with heart conditions as they enter adulthood. Counseling for contraception is stressed with emphasis on decision-making for the contraceptive of choice, their safety, contraindications, and planning for future pregnancies.
Abarbanell et al. [24]	2019	The article summarizes contraceptive safety for women living with a congenital disease of the heart. These females have more risk of pregnancy-related complications, because of which the use of an effective contraceptive is necessary.
Siaplaouras et al. [25]	2023	The study evaluates the role of encouraging children living with congenital heart disease to live a physically active lifestyle. It examines the relationship between these children's physical self-concept and physical activity engagement. The study concluded that these variables relate, implying that these children need a physically active lifestyle.
Gurvitz et al. [26]	2020	The study evaluated adults living with congenital heart diseases of a varied age group through a survey. It highlighted the distribution of this population, comorbidities of both cardiac and non-cardiac origin, and their healthcare use pattern. It provides insight into the need for further such surveys.
Gerardin et al. [27]	2019	The study analyses the percentage of children with congenital disease transferring to adult care from pediatric care. It concluded that individuals with severe disease tend to transfer more to adult healthcare than those with moderate disease.
Insaf et al. [28]	2021	The study evaluates the readiness of individuals living with congenital diseases to receive healthcare in specialized cardiac centers. Based on age, the centers were classified into adult or pediatric. It explores the possible barriers and the levels at which they occur.
Pujol et al. [29]	2022	The study in England explores the transfer rate of children with congenital disease to adult healthcare. It concluded that the transfer rate was efficient, and further initiatives must be taken to target those at increased follow-up risk.
Gurvitz et al. [30]	2023	The study aims to identify the lapses in healthcare, their incidence, and possible barriers in the management of adults living with congenital disease of the heart. These individuals commonly experience interruptions during their late teen years during the transition process and in specific geographic areas for which further research needs to be conducted.
Ricci et al. [31]	2023	The study evaluates the effectiveness of transition clinics to enhance the education of adolescents living with congenital diseases and empower them for a successful transition to adult healthcare. It concluded that patient education positively affected most adolescents and reduced follow-up risk.
Neidenbach et al. [32]	2021	The article assesses the perception of adults living with congenital disease in Germany regarding their utilization of medical care, counseling needs, and perceived satisfaction. These individuals require long-term follow-up for care. However, despite the efforts, the burden of loss of follow-up is still high.
Hager et al. [33]	2005	The article compares the quality of life in adults living with a congenital disease of the heart through cardiac and pulmonary exercises. These tests are considered to check the health status of these patients using various instruments.
Diller et al. [34]	2011	The article discusses the congenital diseases of the heart, highlighting their management and long-term complications. The role of physicians in creating awareness to encourage the utilization of health services through optimal communication is also discussed.
Su et al. [35]	2022	The article assesses the mortality rate in a child living with a congenital disease of the heart through a nationwide study. It is a valuable indicator in both early life and later years, showing increased mortality, leading to questions on the efficacy of their treatment.
Hunter et al. [36]	2016	The article discusses the quality of life of an adult living with a congenital disease of the heart through a cross-sectional study. It compares the quality of life of these adults in various countries using specific tools.
Thomet et al. [37]	2023	This study aims to develop quality indicators for evaluating adolescent congenital heart disease transition programs, which is crucial for assessing and comparing program quality across centers, ensuring uninterrupted and lifelong care.

## Conclusions

There is a noticeable rise in the population of ACHD due to ongoing advancements in diagnosis and treatment. These individuals require specialized care to ensure their long-term well-being. Consequently, an effectively designed transition program requires a multidisciplinary team capable of addressing medical and emotional needs. Active involvement from the patient and their family is crucial to overcome potential barriers that could impede the transition process. They should be guided to understand the program thoroughly and recognize the possible long-term consequences of their actions. Patients need to cultivate skills in self-management and independence to comprehend their condition and practice healthier lifestyles. This collaborative and multidisciplinary process necessitates the cooperation of patients, families, and the ACHD team. The team should be able to cater to the individualized needs of each patient with an emphasis on counseling for healthy sexual practices. The success of this program can be measured through improvements in the quality of life and the extension of life expectancy resulting from the interventions. The global pattern of successful transition and various tools to assess long-term outcomes and patient adherence have been discussed. However, there is a need to develop other reliable indicators that can help improve ACHD care in all parts of the world so that each child with CHD can receive long-term healthcare. 
